# DeepClaw 2.0: A Data Collection Platform for Learning Human Manipulation

**DOI:** 10.3389/frobt.2022.787291

**Published:** 2022-03-15

**Authors:** Haokun Wang, Xiaobo Liu, Nuofan Qiu, Ning Guo, Fang Wan, Chaoyang Song

**Affiliations:** ^1^ Robotics and Autonomous Systems Thrust, System Hub, Hong Kong University of Science and Technology, Hong Kong, Hong Kong SAR, China; ^2^ Department of Mechanical and Energy Engineering, Southern University of Science and Technology, Shenzhen, China; ^3^ School of Design, Southern University of Science and Technology, Shenzhen, China; ^4^ Shenzhen Key Laboratory of Biomimetic Robotics and Intelligent Systems, Department of Mechanical and Energy Engineering, Southern University of Science and Technology, Shenzhen, China; ^5^ Guangdong Provincial Key Laboratory of Human-Augmentation and Rehabilitation Robotics in Universities, Southern University of Science and Technology, Shenzhen, China

**Keywords:** robot learning, data collection, vis-tac sensing, soft robotics, imitation learning

## Abstract

Besides direct interaction, human hands are also skilled at using tools to manipulate objects for typical life and work tasks. This paper proposes DeepClaw 2.0 as a low-cost, open-sourced data collection platform for learning human manipulation. We use an RGB-D camera to visually track the motion and deformation of a pair of soft finger networks on a modified kitchen tong operated by human teachers. These fingers can be easily integrated with robotic grippers to bridge the structural mismatch between humans and robots during learning. The deformation of soft finger networks, which reveals tactile information in contact-rich manipulation, is captured passively. We collected a comprehensive sample dataset involving five human demonstrators in ten manipulation tasks with five trials per task. As a low-cost, open-sourced platform, we also developed an intuitive interface that converts the raw sensor data into state-action data for imitation learning problems. For learning-by-demonstration problems, we further demonstrated our dataset’s potential by using real robotic hardware to collect joint actuation data or using a simulated environment when limited access to the hardware.

## 1 Introduction

Learning from human behaviors is of great interest in robotics (Argall et al., 2009; Kroemer et al., 2019; Osa et al., 2018). Dexterous operation of various tools plays a significant role in the evolution of human behaviors from ancient times (Kaplan et al., 2000) to modern civilization (Brown and Sammut, 2011). For imitation-based manipulation learning, it is common to collect behavior cloning data by directly observing the human hand (Christen et al., 2019) or through human-guided robot demonstration (Chu et al., 2016). However, it is also widely recognized that such dexterity in manipulation is also tightly related to the sense of touch through the fingers (Billard and Kragic, 2019), challenging to model and reproduce with current development in low-cost sensing solutions. For example, recent research in robotic Jenga player (Fazeli et al., 2019) and Gelsight sensors (Yuan et al., 2017) shows the potential in adopting active touch sensing for dexterous manipulation learning. However, a lack of low-cost, efficient, shareable, and reproducible access to the manipulation data remains a challenge ahead.

The correspondence issue with the arm and hand between humans and robots is another challenge yet to be resolved (Dautenhahn and Nehaniv, 2002). The human muscular-skeletal system provides a biological inspiration for modern robot manipulators and grippers, but most are rigidly designed with superhuman bandwidth for industrial purposes with a structural simplification for efficiency and accuracy (Gealy et al., 2019). When training with imitation learning algorithms, the undesirable motions from demonstrations and raw sensory inputs without proper embodiment are also tricky to resolve, given the structural mismatch in arm and hand between the robot learner and human teacher (Osa et al., 2018).

**FIGURE 1:**
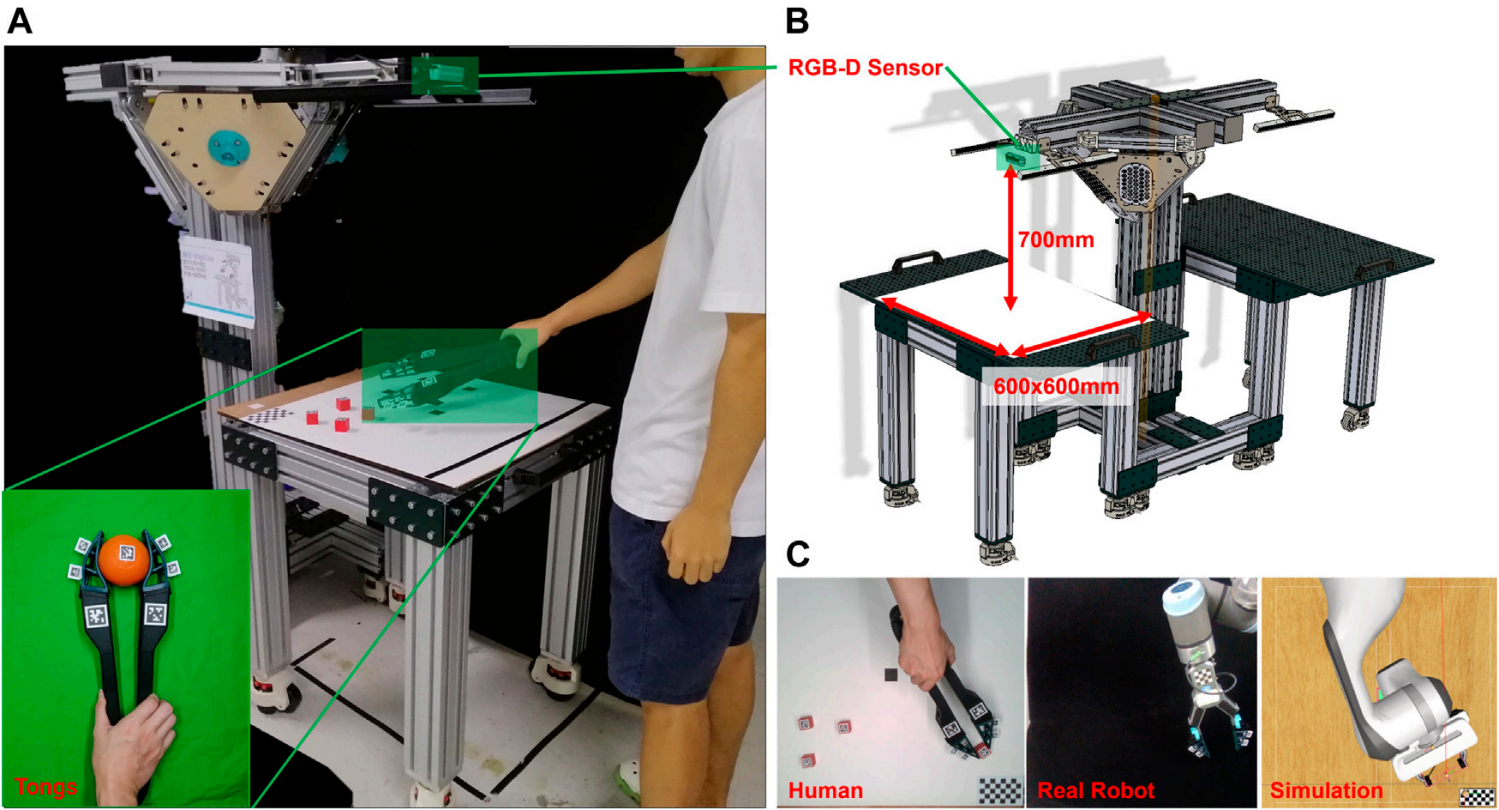
Design overview of DeepClaw 2.0 for collection human manipulation data. As shown in **(C)**: manipulation data collected from a human teacher who operates a pair of modified kitchen tongs can be applied in imitation learning for robot in simulated or real environment. As shown in **(A)** an **(B)**: RGB-D sensor (Intel RealSense 435i is used in this paper) is fixed on the top, 700 mm away from the operating platform. Working space is a square with 600 × 600 mm. The origin of world coordinates is set in the left-top corner located by a checkerboard.

While it is common to learn from human demonstrations for robot learning, it remains difficult to track the dexterous multi-finger motion for exact behavior cloning using 2-finger parallel jaw grippers. Besides direct interaction with objects through the fingers, humans are also skilled at operating tools for dexterous manipulation. In this paper, we propose DeepClaw 2.0 (Figure 1) as a data collection platform for imitation learning, where a human teacher operates a pair of modified kitchen tongs to perform object manipulation. Using a single RGB-D camera (Intel RealSense D435i) as the only sensor, we can track the tongs’ spatial motions with markers. We also attached a soft finger network on each tip of the tongs to infer unified physical interaction with objects using a vision-based force-sensing method. These soft fingers can also be fixed to parallel grippers, bridging the gap of structural mismatch between human fingers and robot grippers for learning. We collected comprehensive data of dexterous human manipulation with tools directly transferable to parallel grippers with the same set of fingers installed by tracking how humans operate the tongs and how the fingers deform. We also demonstrated how researchers could use this platform by feeding the collected data to commercial robot controllers to reproduce the manipulation, generating further data on the actuator angle, velocity, and torque/current incurred on each robot joint. For researchers with limited access to real robot hardware, we also demonstrated the possibility of motion reproduction in a simulated environment using CoppeliaSim (previously known as V-REP). We assumed the manipulation as a Markovian process and converted all data collected to fit a standard Markov Decision Process (MDP) model with an intuitive user interface. The proposed DeepClaw 2.0 platform is open-sourced with all configuration files and data hosted on Github^1^ for shareable and reproducible research, aiming at a low-cost benchmarking solution among the robot learning community.

In the next section, we present the design details of DeepClaw 2.0, focusing on the data collection process. Section 3 presents the experimental design, the collected dataset, and the reproduction in real and simulated robots. Section 4 discusses some of the findings and potentials of the proposed platform. Conclusion, limitations, and future work are enclosed in the final section, which ends this paper.

## 2 The Data Collection Design of DeepClaw 2.0

### 2.1 Design Overview

**FIGURE 2:**
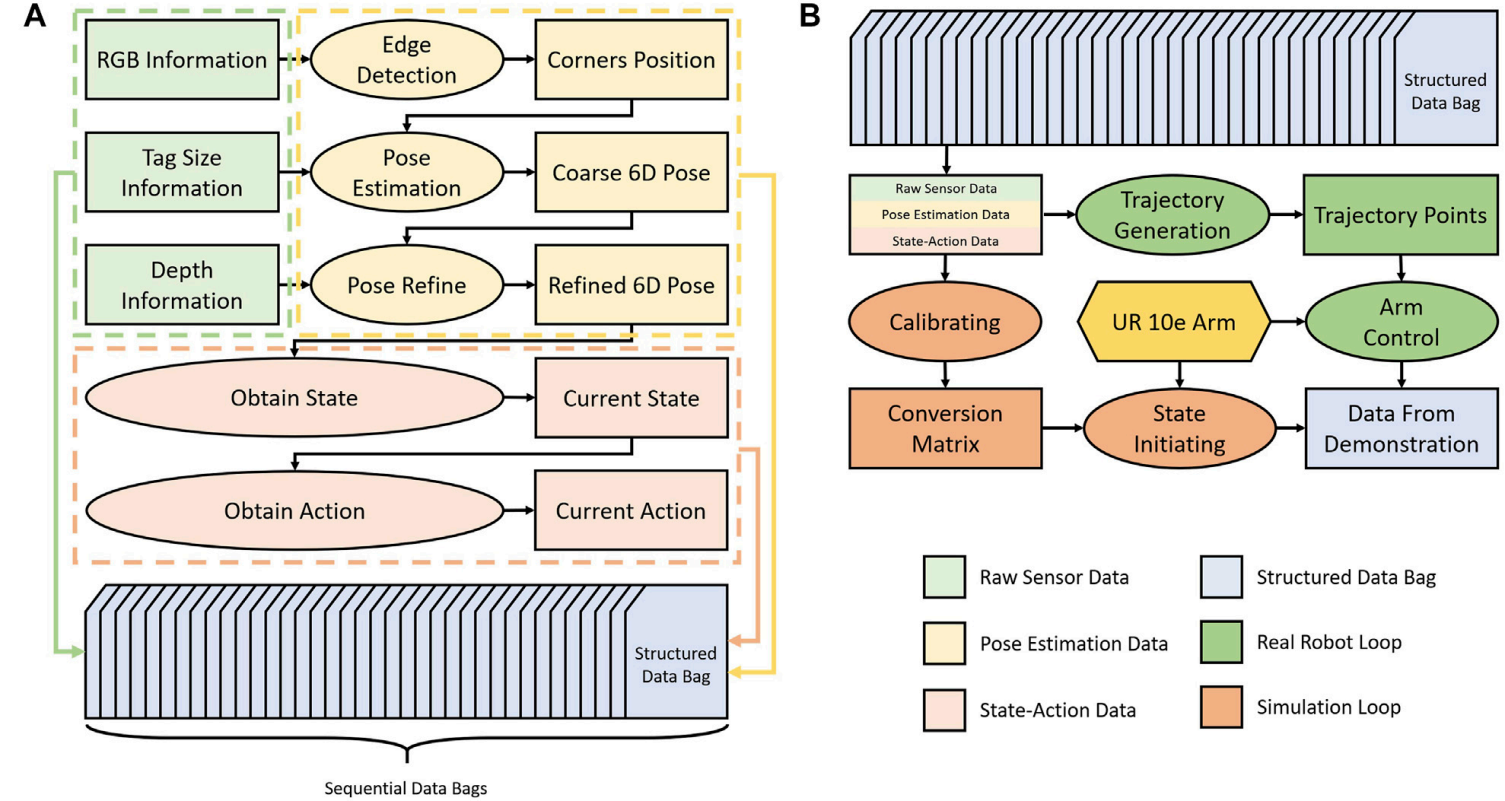
The data collection pipeline of DeepClaw 2.0 is shown in **(A)**, which includes raw sensor data (green), pose estimation data (light yellow), state-action data (orange), and post-processed data (light gray). Raw data consists of three types of information: tag data (ID number and size) as the prior information, RGB image, and depth image captured by a single fixed camera (Intel RealSense D435i in this paper). Low-level features are collected as pose estimation data, include detected corner point and 6D pose of the marker. State-action information, constructed from the above low-level features, reveals the motion of both objects and tongs during the manipulation task. Two typical ways to use structured data bags are shown in **(B)**. The green branch indicates how the real robot reproduces the trajectory recovered from data bags, and the orange branch demonstrates steps to regain manipulation tasks.

The DeepClaw 2.0 aims at providing an open-sourced platform at a low cost to collect human data for learning robotic manipulation. As shown in Figure 2, the platform design leverages: 1) Continuous 6D pose estimation using low-cost cameras and simple markers for all state and action data of tongs, 2) the use of soft finger networks with omni-directional, passive adaptation for unified interaction by the humans and robots, and 3) object manipulation with human operators manually handling a pair of modified, 3D-printable kitchen tongs, which is transferable to parallel 2-finger robot grippers. The collected data involves the following stages of processing before becoming useful for imitation learning or learning-by-demonstration.

• **Raw Sensor Data**: There is only one sensor in DeepClaw 2.0, which is the low-cost Intel Realsense D435i with RGB-D capability. Therefore, the raw sensor data for each example are sequential images *I*_*N*_ = {*i*_0_, *i*_1_, …, *i*_*N*_} of the task environment during the human demonstration, where *i*_*t*_ contains both RGB and depth image, recorded with timestamps *t* ∈ {0, 1, …, *N*}.
• **Pose Estimation Data**: We attach markers on various places of the tongs, fingers, and objects and estimate their 6D poses $P_{N}=\left\{P_{i}^{0},P_{i}^{1},\ldots ,P_{i}^{N}\right\}$ and $\Omega_{N}=\left\{\Omega_{j}^{0},\Omega_{j}^{1},\ldots ,\Omega_{j}^{N}\right\}$ simultaneously by analyzing each recorded image, where $P_{i}^{t},i=1,\ldots ,6$ and $\Omega_{j}^{t},j=1,\ldots ,M$ are poses of markers detected in corresponding image at timestamp *t.* We use $P_{i}^{t}$ to represent pose of marker with ID *i* in the tongs and in the soft finger networks, and $\Omega_{j}^{t}$ to represent pose of marker with ID *j* in objects. The pose vector contains translation and rotation of the marker w. r.t the origin of the world coordinate system.
• **State-Action Data**: Based on the estimated pose data, we can extract information such as the 6D pose of tool center point of the tongs defined at the center of two tags on tongs with an offset to the horizontal plane of the tongs, the gripper’s opening width, and the deformation of soft finger networks. Then, we convert the pose information to state data based on its physical representations. A selected few state data, especially those related to the soft finger networks’ motion and deformation, will be used to extract action data by taking their time derivatives. A suggested way to utilize the pair of state-action data that formulating manipulation learning tasks as a task family described by a distribution of Markov Decision Processes (MDPs), *P*(*M*), which follows the conventions used in (Kroemer et al., 2019). To be more specific, each manipulation *M*_*i*_ is fully defined by a tuple of $\left(S_{r}\times S_{e_{i}},A,R_{i},T_{i},\gamma_{i}\right)$ where *S*_*r*_ is the state of the tongs, including the pose of the *P*_*tcp*_ and three distance variables $\left\|P_{1}^{i}-P_{2}^{i}\|,\|P_{3}^{i}-P_{4}^{i}\|,\|P_{5}^{i}-P_{6}^{i}\right\|$ describing the internal state of the tongs; $S_{e_{i}}$ is the environment state composed of the tabletop setup $S_{w_{i}}$ and the object states $\Omega_{1}^{i}\times \cdots \;,\times \Omega_{j}^{i}$ ; *A* is the action space of the tongs, which is inferred from the state of the tongs; *R*_*i*_ is the reward function depending on the goal of each task; *T*_*i*_ is time horizon i.e., the time steps in each episode; *γ*_*i*_ is the discount factor. The left tuple elements are not explicitly defined or computed.
• **Post-processed Data**: While one can already make use of the above state-action data for learning, we further integrated the capability for data reproduction and collection with real and simulated robots for joint and actuator data, which can be used for demonstration-related learning algorithm development (Argall et al., 2009).

### 2.2 Hardware Mechanic Design

**FIGURE 3:**
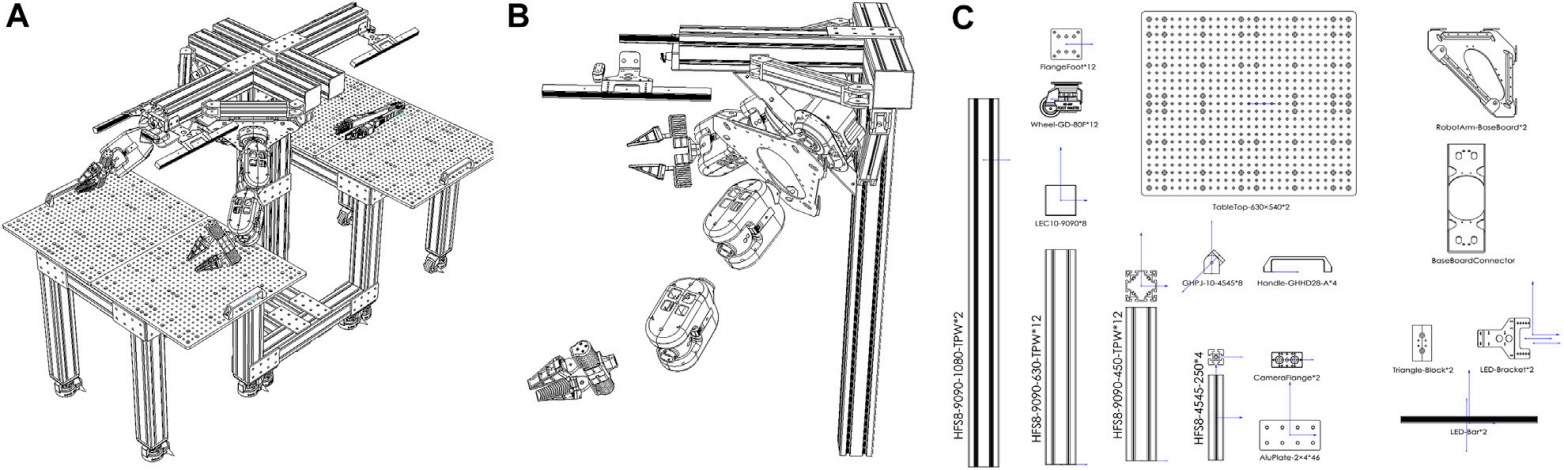
DeepClaw2.0 Station: **(A)** 3D view, **(B)** robot arm explosion view, **(C)** details about components for data collection.

As a shareable and reproducible standardized robot cell, the mechanical frame of DeepClaw2.0 uses aluminum extrusions easily obtained from local shops or global suppliers such as MISUMI Group Inc. In order to achieve ease of assembly, the DeepClaw2.0 platform uses aluminum plates that are drilled with a 2-by-4 hole array, and these aluminum plates are easily machined from local shops. Using bolts and connectors to assembly aluminum extrusions and drilled plates is convenient. Other components such as flanges and wheels are also needed and easy to assembly. All the components are listed in the Figure 3 and Table 1, and more details are shown in Supplementary Figure S1. In order to provide a better light environment for data collection using cameras, we added two lighters fixed by plates. Also, we use some machined plates as the base of robot arms, which show the potential of DeepClaw2.0 as a data collection platform and a system that can realize the process from data collection to use. The top-left corner of Figure 3 shows the detail of the robot arms, which using DeepClaw as a base to perform tasks such as implementing a trained learning model. The center and top-right of Figure 3 show the ability of DeepClaw to collect data and use data simultaneously. The bottom-right of Figure 3 shows the details of the gripper for data collection.

**TABLE 1:** Bill of materials for DeepClaw 2.0.

Number	Type	Quantity	Number	Type	Quantity
1	AluExtru:HFS8-9090-1080-TPW	2	10	CameraFlange	2
2	AluExtru:HFS8-9090-630-TPW	12	11	Handle-GHHD28-A	4
3	AluExtru:HFS8-9090-450-TPW	12	12	TableTop-630 × 540	2
4	AluExtru:HFS8-4545-250-TPW	4	13	RobotArm-BaseBoard	2
5	AluExtruCover:LEC10-9090	8	14	BaseBoardConnector	1
6	Wheel-GD-80F	12	15	Triangle-Block	2
7	FlangeFoot	12	16	LED-Bracket	2
8	GHPJ10-4545	8	17	LED-Bar	2
9	AluPlate-2×4	46	—	—	—

### 2.3 From Raw Sensor Data to Pose Estimation Data

The raw sensor data consist of color, and depth images with timestamps streamed at 30 fps from Intel Realsense D435i. Most researchers utilized some optical motion tracking system to extract human manipulation data (Kolahi et al., 2007). Commercialized high-precision products usually involve a multi-camera setup and complex system configuration, becoming expensive for shareable and reproducible research.

Pose estimation and tracking are the key factors affecting the performance of a motion capture system. We compared three commonly used marker detection algorithms using the same Realsense D435i camera. In the experiments, a 3D-printed L-shape board was mounted on the tool flange of UR10e. A 3 *cm* × 3 *cm* ArUco tag, a 4 × 5 checkerboard with grid size 0.6 cm, and a 3 *cm* × 3 *cm* AprilTag were attached to the L-shape board’s center, respectively. UR10e repeated the same trajectory while we tracked the markers. We sampled ten fixed waypoints along the trajectory and calculated the detection success rate, the average computation time, and the average rotational error (Suzui et al., 2019), as shown in Table 2. The trajectory was repeated ten times for each marker. Our results show that the AprilTag detector in ViSP is the most reliable method among the three, with a 100% detection success rate with the least rotational error at 0.36 radian. It also performed consistently in terms of efficiency with the least variations. The AprilTag detector in ViSP is the only method utilizing the extra depth information provided by the Realsense D435i, which might contribute to its superior performance among the three methods tested.

**TABLE 2:** Marker comparison for 6D pose estimation.

Method	Detection success rate (%)	Average computation time (s)	Average rotational error (rad)	Average position error (cm)
Aruco Minichino (2015)	32	**0.16 (0.17)**	1.60 (0.03)	0.292 (0.162)
Checkerboard Minichino (2015)	90	0.42 (0.12)	0.96 (0.19)	1.005 (0.628)
AprilTag in ViSP (Marchand et al. (2005)	**100**	0.20 (0.01)	**0.36 (0.12)**	**0.085 (0.057)**

Bold values represent the better performance.

**FIGURE 4:**
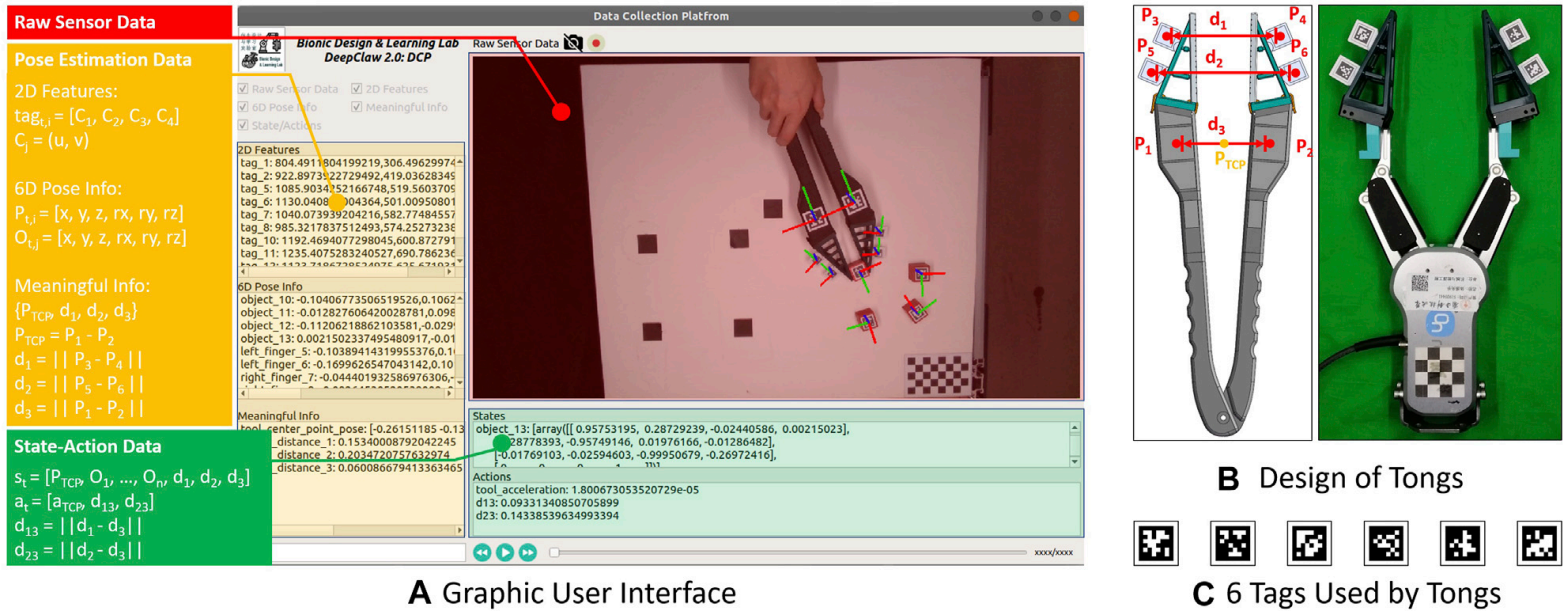
Key components in human manipulation: **(A)** Shows the rendered graphic user interface in DeepClaw 2.0, consists of real-time RGB data flow (highlighted by the red rectangle), low-level features (highlighted by the yellow rectangle), and high-level state-action information (highlighted by the green rectangle). **(B)** Shows the similarities between the assembled tongs and an OnRobot RG6 gripper, some key parameters are pointed, and in **(C)**, six tags from the AprilTag 36h11 family used in this paper are shown.

As a result, we adopted the AprilTag as the marker for our modified tongs design, as shown in Figures 4B,C. A set of the soft finger networks with omni-directional adaptation is also adopted, with further technical details explained in (Yang et al., 2020a,b; Wan et al., 2020). Both the tongs and soft finger networks are 3D printable. Three tags are fixed on each arm of the tongs, with one is near the end of the arm and two attached to the back of each soft finger network. All six tags can be uniquely identified and localized, producing a set of marker poses $P_{i}^{t}$ where *i* = 1, …6 and *t* is the time sequence. The tags were also attached to the objects so that we can keep track of the object poses $O_{j}^{t}$ where *j* iterates over the object ID.

We also developed an intuitive graphical interface, as shown in Figure 4A, which can be used to visualize the real-time data streaming during the data collection as well as to examine the collected data.

### 2.4 Deformation Estimation *via* Passive Perception

It is essential and meaningful to estimate the contact behavior during manipulation. For example, in the task of grasping soft objects or objects of unknown weight, data generated by the interaction between humans and the environment during the teaching process can provide the robot with more abundant information, such as the closing distance of gripper or force adjustment strategy (Yuan et al., 2015). The tactile sensor can provide interactive information, which is ignored by the camera, for robot operations. However, the formation of data provided by the different tactile sensors is various and highly related to its mechanical design. It is not easy to generalize tactile sensing across different types of sensors. To fill this gap, DeepClaw 2.0 uses a low-cost RGB-D camera and provides a visual detection algorithm to estimate the contact deformation of the soft finger network passively. By capture the motion of markers that are fixed at the finger, the contact behavior is recognized. We conducted experiments on ten typical manipulation tasks and compared the deformation of soft finger networks when interacting with objects of different shapes, such as cube and orange, and objects made of soft or solid materials, such as sponge and can.

### 2.5 Optional Collection of Post-processed Data

To verify the reproducibility of the structured data collected by DeepClaw 2.0, we transfer the collected data into the controller of a physical robot arm and a simulation environment. This post-processing step enables us to obtain robot state data about the robot joint/actuator. Based on the action information in collected data, we feed the smoothed trajectories in Cartesian space generated from human manipulators to the physical robot arm. The robot arm obtains the inverse kinematic solution and executes the motion planning by shadowing the human manipulations. The exact reproduction process can be done with simulated robot arms. Thus, the data can be used for robot learning-by-demonstration of specific tasks in the real world and in simulation.

## 3 Experiments and Results

### 3.1 Experiment Setup and Procedure

**FIGURE 5:**
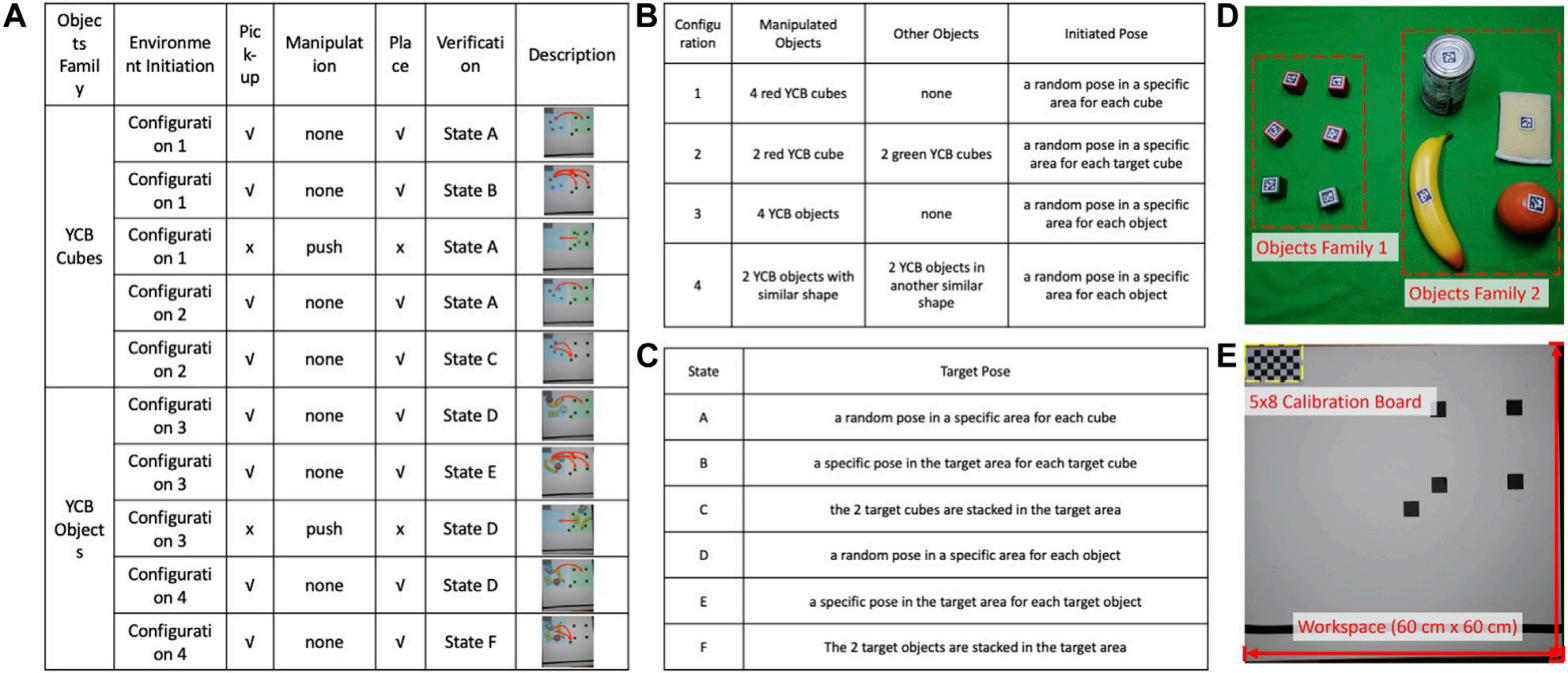
We defined 10 manipulation tasks shown in **(A)**. This paper uses two objects families: four red and two green wooden cubes as one set and four common objects from YCB as another set. We stress two states and three operations in one manipulation task: initial state, target state, picking, pushing, and placing. The details of different initial states and target states are listed in **(B,C)**. The two families of objects from YCB are shown in **(D)**: A set of six cubes; a set of the orange, banana, sponge, and can. The setup of tabletop is shown in **(E)**.

We divide a manipulation task into five phases: The initial state, picking, manipulation, placing, and target state, where manipulation refers to robot actions other than picking and placing. By exploring the setup of objects and whether to execute picking, placing, or other manipulations, we designed a family of ten tasks, as summarized in Figure 5 and Supplementary Table S1. From the Yale-CMU-Berkeley Object and Model Set (Calli et al., 2015), we selected the small wooden cubes of different colors and a few sample objects to represent two levels of object complexity. Each object is attached with a unique AprilTag for pose estimation. For each object set, the five tasks in Figure 5A from left to right differ in manipulation complexity. The origin of the world coordinate is at the bottom-right corner of the checkerboard with the *z*-axis facing upward. All the collected pose data are transformed relative to the world coordinate.

We invited five operators, asking each to repeat each of the ten tasks in five trials. As a result, we collected a dataset of 250 task executions in total. Each task execution data consists of a sequence of color and depth images, one pack of a structured data bag, and one video of the manipulation process recorded by another camera. The researcher starts the experiment by launching the data visualization interface. The operator is asked to stand in front of the tabletop holding the tongs in the right hand. After receiving the starting signal from the researcher, the operator starts executing the manipulation task. Once completed, the operator notifies the researcher to stop the recording in the user interface.

### 3.2 Experiment Results

**FIGURE 6:**
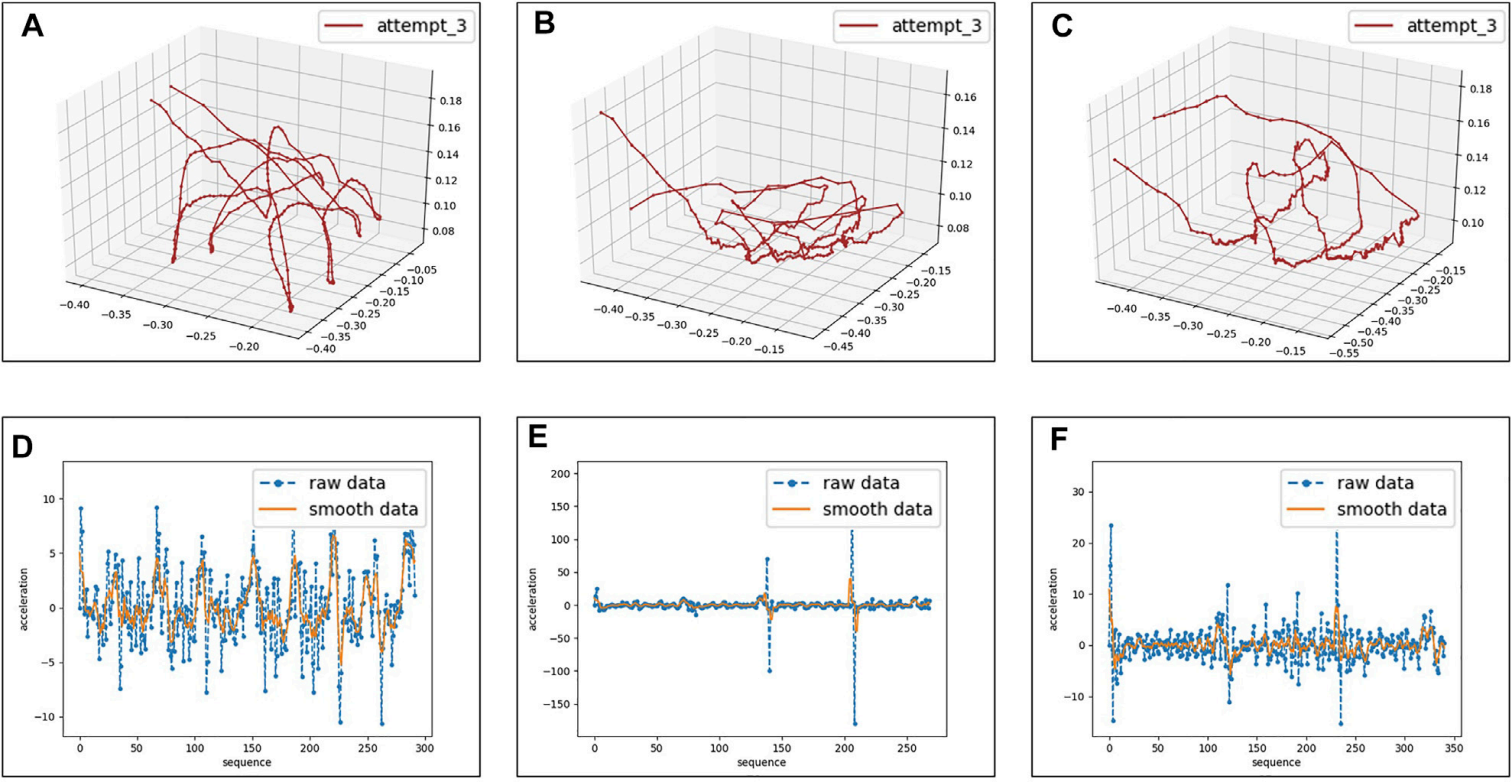
Experiment results and analysis of the collected data. We plot the trajectory of the third attempt of task 2, task 3, and task 8 from a human operator in **(A–C)**. Operations (pushing, picking, or placing) and (initial or target) state can be easily distinguished by observing the motion trajectory. Sub-figures from **(D)** to **(F)** at the bottom line represent the corresponding acceleration sequence with and without smoothing. Motion-related data, such as position, velocity, and acceleration, provides a quantifiable indicator of operations.

We performed a mean filter to reduce the disturbance during the data collecting process. A sample statistical overview of the data is visualized in Figure 6. The statistical analysis provides a glimpse of the characteristics in each manipulation task, where different phases of a single task can be clearly identified. The trajectory visualization provides support for the reproduction experiment in real and simulated robots.

### 3.3 Experiment With Real and Simulated Robots

We reproduced the trajectory of a human operator executing task 1. Then, do the reproduction with UR10e. The robot states, including joint angle, joint current, and joint velocity, were recorded during the process. These experiments show that the structured data collected from different manipulation tasks can be reproduced by the robot arm in the same data process.

We also reproduced the same task in simulation using CoppeliaSim (Freese et al., 2010). The object’s pose information was utilized to render an initial state of the objects cloning the real scene. The simulated Franka Emika arm with its 2-finger gripper then tried to follow the trajectory of a human operator. As shown in Figure 1, the simulated robot can reproduce task 1 recorded with a human operator. Please refer to the Github page for further details on the motion reproduction.

## 4 Discussion

### 4.1 Dexterous Object Manipulation Through Operating Tools

Tracking human hand motion can be challenging and expensive (Billard and Kragic, 2019). Recent research already demonstrated success in tracking refined and real-time hand motion for training advanced manipulation skills using physical (OpenAI et al., 2019) or simulated robots (Han et al., 2018). Besides a multi-camera motion capture system, data gloves are another alternative (Sundaram et al., 2019; Dillmann, 2004), but may suffer from interfering with the natural motion and touch feeling of the human hand. Even when such data becomes cost-effectively available, which is yet to be the case, there remains another challenge in the availability of robotic hands matching the human’s capability at a lower cost. While many multi-fingered robot hands (OpenAI et al., 2019; Zhou et al., 2019) are available, there remains a gap between technological maturity and cost-effectiveness.

This paper proposes a different perspective by collecting large-scale object manipulation data when human teachers are operating tools. Specifically, we adopt tongs as the tool of interest, commonly found in life and workplaces for food preparation or material handling. A convenient feature of the tongs is its two-fingered design, which is structurally similar to robotic grippers with parallel fingers. Although most tongs adopt a pivot or scissor mechanism, they usually come with “long arms terminating in small flat circular ends of tongs^2^,” resulting in a convenient approximation to a parallel motion at a low cost. Our results demonstrate the potential to collect dexterous manipulation data from such configuration, which can be translated for imitation learning at a low cost in data collection.

### 4.2 Vision-Based Force Sensing Using Soft Finger Network

We introduce a pair of soft finger networks to generate pseudo-force data about the physical interaction between the tongs and objects to keep the system simple, cost-effective, and versatile in data collection. Such a soft finger network provides an overconstrained grasping solution through compliant interaction at a low-cost (Yang L. et al., 2020; Wan et al., 2020), which can be leveraged through optical methods for non-contact force-sensing after calibration. Recent research demonstrated the possibility of embedding optical fibers inside such a simple finger network for tactile sensing in grasping (Yang Z. et al., 2020).

**FIGURE 7:**
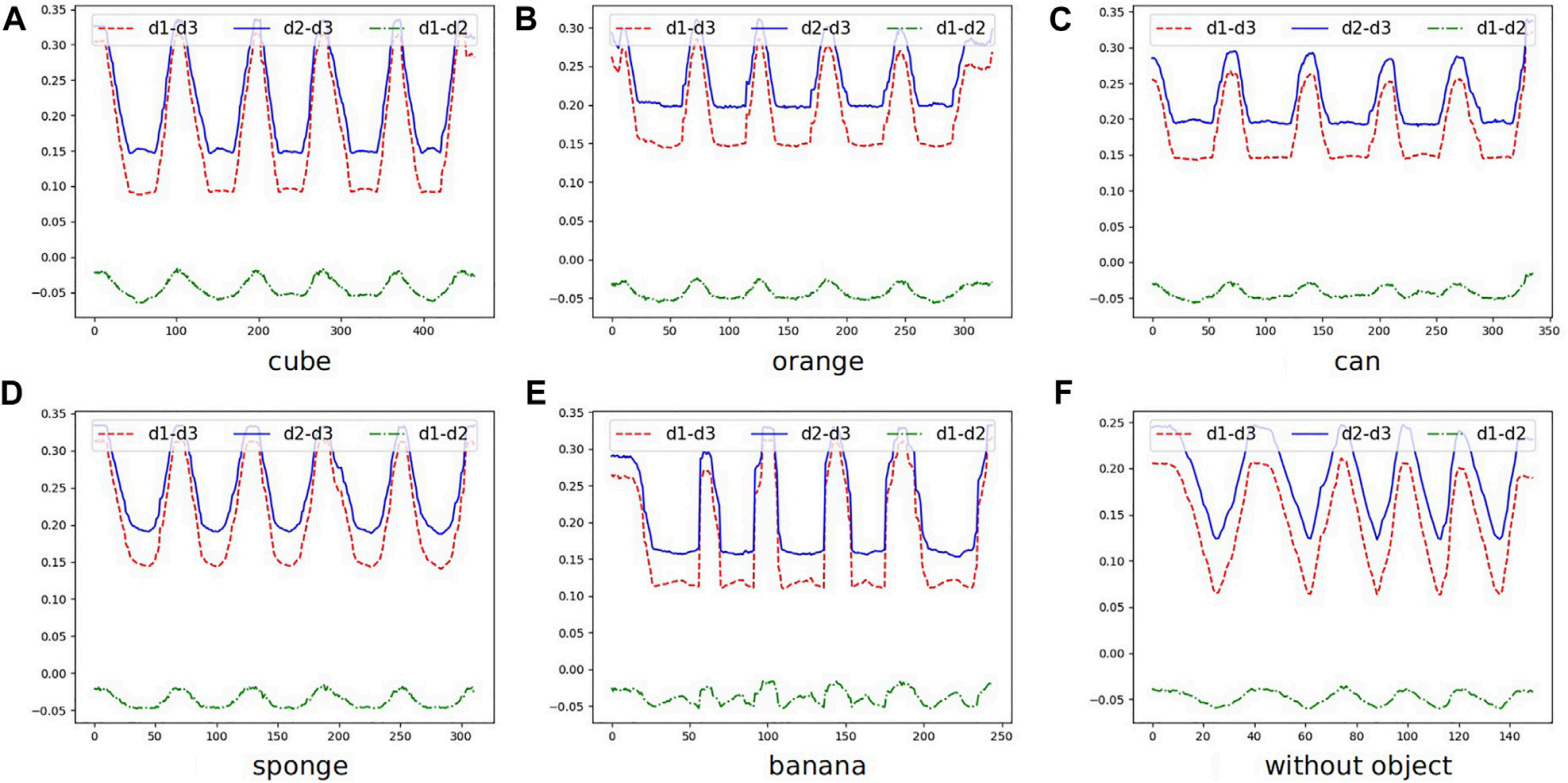
The contact information of grasping five objects(**(A)**: cube, **(B)**: orange, **(C)**: can, **(D)**: sponge, and **(E)**: banana) and no object **(F)**) five times. To emphasis the differences in interaction, we record subtraction of every two values of distance. d1 and d2 reveal the deformation of soft finger networks. d3 is linear and proportional to the closing distance of tongs. Therefore, the subtraction of d1 and d3, or d2 and d3, may provide information of geometry (lower value when grasped means smaller size of the object) and material (curve or straight line at valley reveals soft or solid material). The precise prediction will be exploited in the future.

In this paper, we use markers instead of unifying the raw sensory data in image format and tracking the markers to collect the motion and deformation data of the soft finger when interacting with the objects, as shown in Figure 7. When both the robot learner and the human teachers use the same soft finger networks, we can directly use the deformation of the fingers as the pseudo-force data of physical interaction. If necessary, we can calibrate the soft finger network’s stiffness to calculate the specific interaction force based on the measured deformation (Liu, 2020; Yang, 2020). Since these soft finger networks can be conveniently installed on the arms of the tongs and the robotic grippers, we can maintain a consistent interface of physical interaction. Such a vision-based sensory mechanism holds the potential to reduce the hardware cost and system complexity significantly, yet maintaining a consistent level of data robustness and conformity for training and deployment.

### 4.3 Feasibility of the Collected State-Action Data

The primary purpose of the proposed DeepClaw 2.0 platform is to facilitate the large-scale data collection for training models for manipulation learning algorithms, which is usually fitted to an MDP model. The DeepClaw platform facilitates a streamlined pipeline that encodes the sequential Raw Sensory Data in time-series image formats into State-Action Data with physical meanings for manipulation learning. Researchers can directly use State-Action Data by introducing specific transition functions, reward functions, and discount factors to develop algorithms based on MDP models for imitation learning.

For researchers interested in learning-by-demonstrations, if a physical robot is available, we also demonstrated the feasibility of collecting actuator data in motor current and joint data in angular position and velocity by feeding the collected State-Action Data to the controllers using a UR10e robot. Even if robot hardware is unavailable, we also demonstrated the feasibility of using a simulated Franka Emika robot to achieve a similar purpose, which may suffer from a reduced richness in data variety, but an enhanced benefit in reproducing the task with variant objects.

## 5 Conclusion

In this paper, we proposed the DeepClaw 2.0 platform with a low-cost RGB-D camera to collect the training data of object manipulation by tracking the spatial motion and deformation of a pair of specially designed soft finger networks with omni-directional adaptation. These fingers are used as a universal agent of physical interaction for dexterous objects manipulation, either by humans when operating a pair of tongs fixed with these fingers or by a robot with a common parallel jaw-gripper with such fingers installed. We can collect the pseudo-force interaction data between the fingers and objects using vision-based force-sensing through these soft finger networks. We presented an intuitive interface to manage the State-Action Data collection process for training imitation learning algorithms. We demonstrated a viable solution to collect further robot state and action data, which can be alternatively used for research on learning-by-demonstration.

This study is limited to a performance benchmark of the collected data for training manipulation learning, which will be studied in future work. As the RGB-D camera is the only sensor used, the quality of the collected data relies heavily on the performance of the camera used (Intel RealSense D435i). If higher resolution is necessary, systems such as Photoneo MotionX (Wan and Song, 2020) could be a potential alternative for high-quality point clouds with gray-scaled images, but at a much higher price. The vision-based force-sensing capability can be further improved for sensitivity and accuracy. We are currently working on an alternative design by embedding cameras inside the soft finger for more sensitive sensing, which will be addressed in another paper.

The future work of this study mainly aims at a more comprehensive system design towards a low-cost, open-sourced platform for robot manipulation learning research. As shown in Figure 1 right, the full design of DeepClaw 2.0 also involves a second robot station with a lobster-inspired robot with soft fingers on a parallel gripper. Recent research by the Berkley Open Arm project (Gealy et al., 2019) proposed a promising design paradigm in this direction for robot hardware that “enables useful automation in unconstrained real-world human environments at low cost”.

## Data Availability

The datasets presented in this study can be found in online repositories. The names of the repository/repositories and accession number(s) can be found below: https://github.com/bionicdl-sustech/DeepClaw.

## Footnotes

1
https://github.com/bionicdl-sustech/DeepClaw.

2
https://en.wikipedia.org/wiki/Tongs.
