# Supplementary material for: DeepClaw 2.0: A Data Collection Platform for Learning Human Manipulation
Source: Front Robot AI. 2022 Mar 15;9:787291. doi: 10.3389/frobt.2022.787291 (PMC8964492; doi:10.3389/frobt.2022.787291)
Supplement: Supplementary file 1 [file DataSheet1.PDF]

# Supplementary Material

## 1 COMPONENTS OF DEEPCLAW 2.0

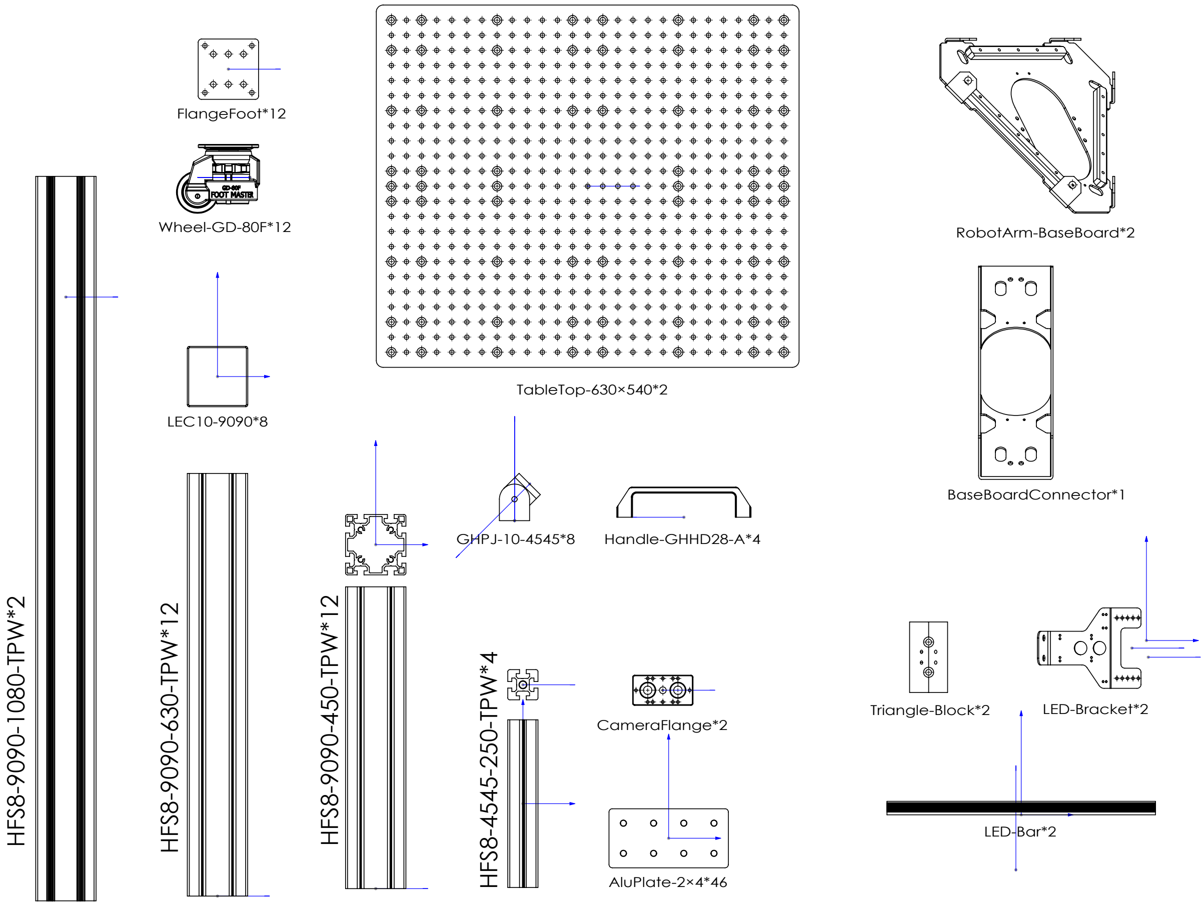


**Figure S1.** All components of the DeepClaw 2.0 station.

## 2 TASK DESCRIPTIONS AND RESULTS

**Table S1.** The descriptions, schematic diagrams and trajectories of 10 tasks. The trajectory results are positions of the left tag on tongs during five task attempts.

| Task | Description | Schematic Diagrams | Trajectory Result |
|---|---|---|---|
| Task1 | Pick 4 red cubes from a random pose in the initial area for each cube to a random pose in the target area for each cube | 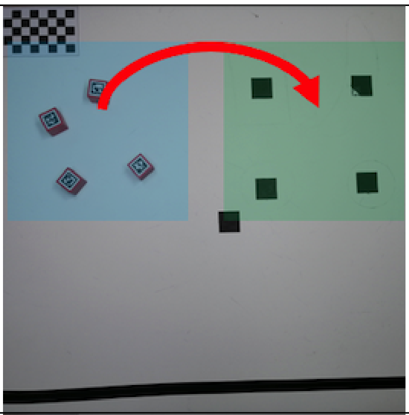 | 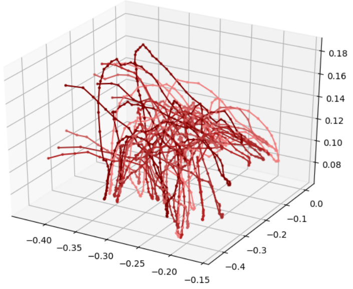 |
| Task2 | Pick 4 red cubes from a random pose in the initial area for each cube to a specific pose in the target area for each cube | 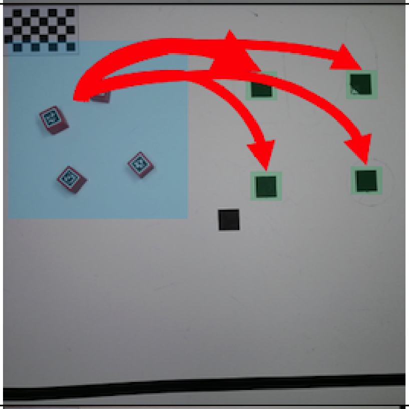 | 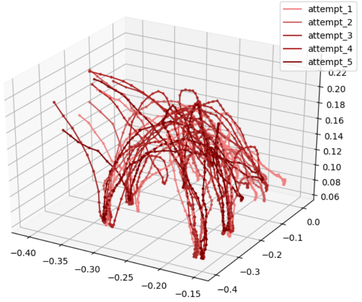 |
| Task3 | Push 4 red cubes from a random pose in the initial area for each cube to a random pose in the target area for each cube | 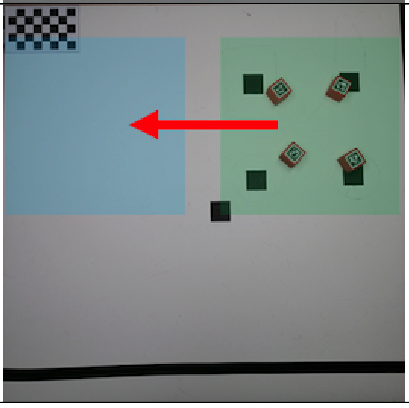 | 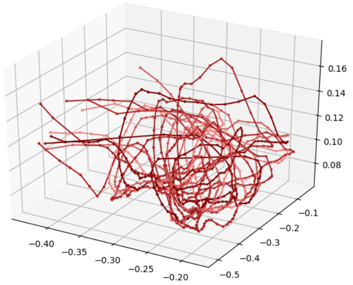 |

| | | | |
|---|---|---|---|
| Task4 | Pick 2 red cubes as target objects with 2 green cubes as other objects from a random pose in the initial area to a random pose in the target area | 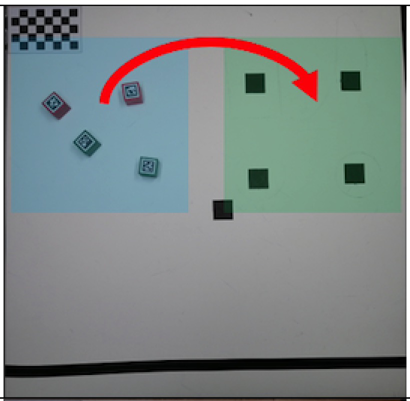 | 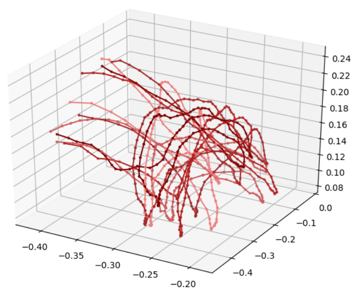 |
| Task5 | Pick 2 red cubes as target objects with 2 green cubes as other objects from a random pose in the initial area to the state in which the 2 target cubes are stacked in the target area | 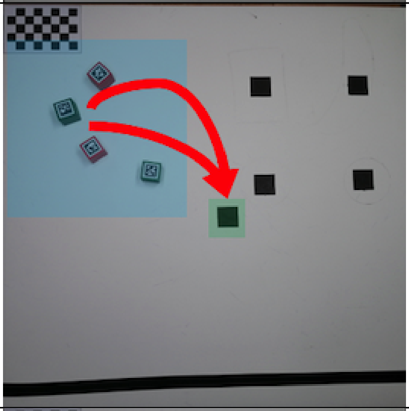 | 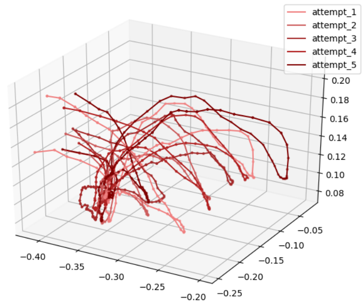 |
| Task6 | Pick 4 YCB objects from a random pose in the initial area for each object to a random pose in the target area for each object | 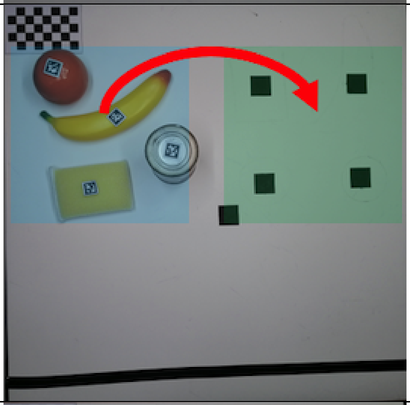 | 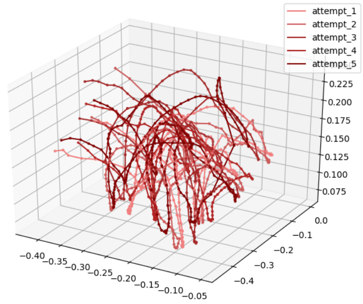 |
| Task7 | Pick 4 YCB objects from a random pose in the initial area for each object to a specific pose in the target area for each object | 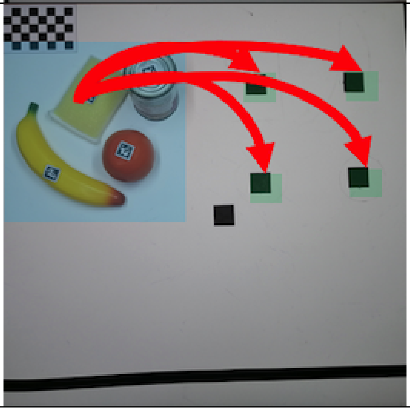 | 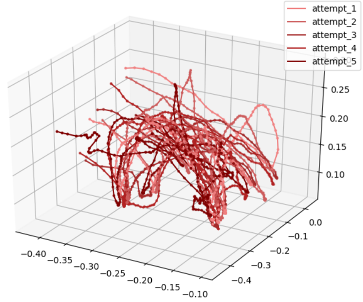 |

| | | | |
|---|---|---|---|
| Task8 | Push 4 YCB objects from a random pose in the initial area for each object to a random pose in the target area for each object | 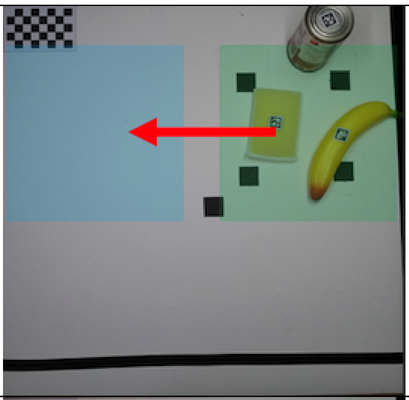 | 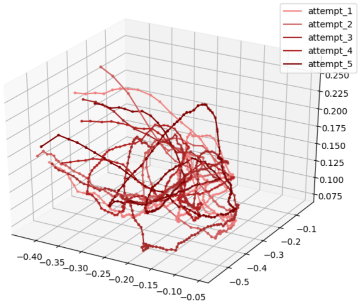 |
| Task9 | Pick 2 specific YCB objects as target objects with another 2 YCB objects as other objects from a random pose in the initial area to a random pose in the target area | 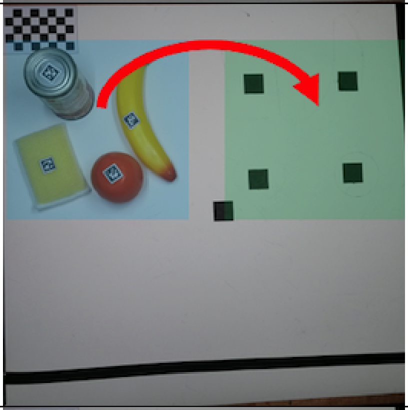 | 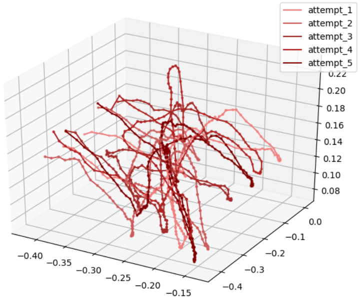 |
| Task10 | Pick 2 specific YCB objects as target objects with another 2 YCB objects as other objects from a random pose in the initial area to the state in which the 2 target objects are stacked in the target area | 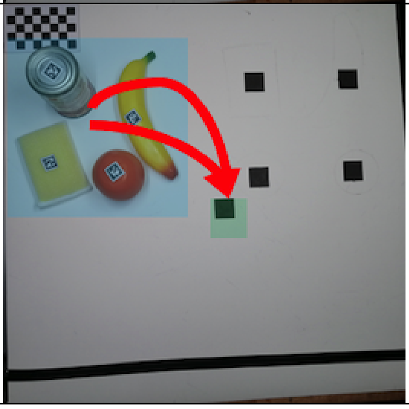 | 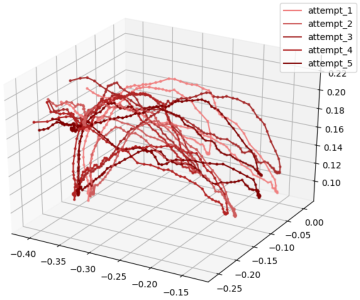 |
